# Parameter correlations maintaining bursting activity

**DOI:** 10.1186/1471-2202-15-S1-O9

**Published:** 2014-07-21

**Authors:** Anca Doloc-Mihu, Ronald L Calabrese

**Affiliations:** 1 Department of Biology, Emory University, Atlanta, GA, 30322, USA

## 

In this study, we focused on the role of correlated conductances in the robust maintenance of functional bursting activity. Recent experimental and computational studies suggest that linearly correlated sets of parameters (intrinsic and synaptic properties of neurons) allow central pattern generating (CPG) neurons to produce and maintain their rhythmic activity regardless of changing internal and external conditions. However, the mechanisms that allow multiple parameters to interact, thereby producing and maintaining rhythmic network activity, are less clear.

**Figure 1:**
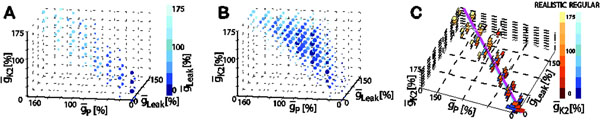
Plots of the groups of instances in the 3D space given by the ḡ_Leak_ , ḡ_K2_, and ḡ_P_ maximal conductances. **A**. Realistic isolated neurons (83 points; 307 instances); **B**.Realistic HCOs (243 points; 99,066 instances); **C**. Realistic and regular/ not realistic isolated neurons (83 realistic vs. 91 regular points) and their ODR lines, magenta for realistic and cyan for regular.

For our study, we used our existing database (HCO-db) [1] of instances of a half center oscillator (HCO) model [2]. The HCO single-compartment conductance-based model [2] consists of two mutually inhibitory neurons and replicates the electrical activity of the oscillator interneurons of the leech heartbeat CPG under a variety of experimental conditions. From the database, we identified functional activity groups of isolated neuron and half-center oscillator (HCO) model instances and realistic subgroups of each such group that showed burst characteristics (principally period and spike frequency) similar to the animal. To find linear correlations among the conductance parameters maintaining functional leech bursting activity, we applied Principal Component Analysis (PCA) to each of these four groups. PCA identified a set of three maximal conductances (leak current, ḡ_Leak_; a persistent K current, ḡ_K2_; and a persistent Na^+^ current, ḡ_P_) that correlate linearly for the two groups of regular and realistic isolated neuron instances (Figure 1 A). Our 3D visualizations of HCO instances (Figure 1 B) in the reduced space of ḡ_Leak_ , ḡ_K2_, and ḡ_P_ suggested that there might be a non-linear relationships between parameters for these instances.

A least square fit regression line (3D Orthogonal Distance Regression (ODR) line) to each group of isolated neurons (Figure 1 C) showed a tendency for the realistic instances to be at the high values on all axes and a tendency of the regular/not realistic instances to be at the low and middle values on all axes. From our analysis, it appears that none of the ḡ_Leak_ , ḡ_K2_, or ḡ_P_ parameters is sufficient by itself to produce regular and realistic isolated neuron instances, but they must work together (in linear combination) in almost equal amounts towards producing the respective instances. Experimental studies have shown that period is a key attribute influenced by modulatory inputs and temperature variations in heart interneurons. Thus, we explored the sensitivity of period to changes in maximal conductances of ḡ_Leak_, ḡ_K2_, and ḡ_P_, and we found that for our realistic isolated neurons the effect of these parameters on period could not be assessed because when varied individually bursting activity was not maintained. Current studies are focused on determining which parameters can, when varied, smoothly control period, while maintaining bursting activity.
